# IRE1α Is a Therapeutic Target for Cystic Fibrosis Airway Inflammation

**DOI:** 10.3390/ijms22063063

**Published:** 2021-03-17

**Authors:** Emily A. Hull-Ryde, John T. Minges, Mary E. B. Martino, Takafumi Kato, Jacqueline L. Norris-Drouin, Carla M. P. Ribeiro

**Affiliations:** 1 Marsico Lung Institute and Cystic Fibrosis Research Center, School of Medicine, University of North Carolina, Chapel Hill, NC 27599, USA; hullryde@email.unc.edu (E.A.H.-R.); john_minges@med.unc.edu (J.T.M.); marybmartino@gmail.com (M.E.B.M.); taka_kato@med.unc.edu (T.K.); 2 Center for Integrative Chemical Biology and Drug Discovery, Eshelman School of Pharmacy, University of North Carolina, Chapel Hill, NC 27599, USA; jnorris@unc.edu; 3 Division of Pulmonary Diseases, Department of Medicine, School of Medicine, University of North Carolina, Chapel Hill, NC 27599, USA; 4 Department of Cell Biology and Physiology, School of Medicine, University of North Carolina, Chapel Hill, NC 27599, USA

**Keywords:** cystic fibrosis, airway epithelia, airway inflammation, cytokine, unfolded protein response, inositol requiring enzyme 1α, KIRA6, CFTR modulators

## Abstract

New anti-inflammatory treatments are needed for CF airway disease. Studies have implicated the endoplasmic reticulum stress transducer inositol requiring enzyme 1α (IRE1α) in CF airway inflammation. The activation of IRE1α promotes activation of its cytoplasmic kinase and RNase, resulting in mRNA splicing of X-box binding protein-1 (XBP-1s), a transcription factor required for cytokine production. We tested whether IRE1α kinase and RNase inhibition decreases cytokine production induced by the exposure of primary cultures of homozygous F508del CF human bronchial epithelia (HBE) to supernatant of mucopurulent material (SMM) from CF airways. We evaluated whether IRE1α expression is increased in freshly isolated and native CF HBE, and couples with increased XBP-1s levels. A FRET assay confirmed binding of the IRE1α kinase and RNase inhibitor, KIRA6, to the IRE1α kinase. F508del HBE cultures were exposed to SMM with or without KIRA6, and we evaluated the mRNA levels of XBP-1s, IL-6, and IL-8, and the secretion of IL-6 and IL-8. IRE1α mRNA levels were up-regulated in freshly isolated CF vs. normal HBE and coupled to increased XBP-1s mRNA levels. SMM increased XBP-1s, IL-6, and IL-8 mRNA levels and up-regulated IL-6 and IL-8 secretion, and KIRA6 blunted these responses in a dose-dependent manner. Moreover, a triple combination of CFTR modulators currently used in the clinic had no effect on SMM-increased XBP-1s levels coupled with increased cytokine production in presence or absence of KIRA6. These findings indicate that IRE1α mediates cytokine production in CF airways. Small molecule IRE1α kinase inhibitors that allosterically reduce RNase-dependent XBP-1s may represent a new therapeutic strategy for CF airway inflammation.

## 1. Introduction

Cystic fibrosis (CF) pulmonary disease is the culmination of a series of events consequential to mutations in the CF transmembrane conductance regulator (CFTR). Defective CFTR function results in decreased Cl^−^ secretion associated with alterations in sodium transport [1,2], resulting in airway dehydration [3], thickened mucus that accumulates on the airway surface, and impairment of mucociliary clearance [4,5,6]. These alterations lead to persistent bacterial infection-induced chronic inflammation [7,8,9,10,11,12,13,14,15], which can harm the airways of CF patients [16]. Indeed, it has been suggested that the CF airway inflammatory response is “excessive” [17,18].

The specific inflammatory response of CF airway epithelia is a key contributing factor for the chronically inflamed status of CF airways [19,20,21]. For instance, CF airway epithelia exhibit persistent activation of nuclear factor-κB (NF-κB), elevated production of pro-inflammatory cytokines, and decreased secretion of anti-inflammatory mediators [22,23,24,25]. We have shown that inflammation of airway epithelia promotes expansion of endoplasmic reticulum (ER) Ca^2+^ stores [26], which can contribute to airway inflammation via amplification of Ca^2+^-mediated cytokine production [22]. This airway epithelial response is likely beneficial for acute infection in airways with normal mucociliary clearance by helping to clear the infectious insult. In contrast, in obstructed CF airways, the amplification of Ca^2+^-dependent inflammatory responses resulting from the expansion of ER Ca^2+^ stores is likely ineffective in improving the clearance of chronic infection in thickened mucus and could damage the airway walls [22,27].

Increased protein synthesis, including up-regulation of cytokine production resulting from CF airway epithelial inflammation, triggers ER stress, which activates the unfolded protein response (UPR) [28,29,30]. In mammalian cells, the UPR is comprised by three major pathways: (1) inositol requiring enzyme 1 (IRE1), (2) activating transcription factor 6 (ATF6), and (3) PKR-like ER kinase/pancreatic eIF2α kinase (PERK). Because our previous studies have revealed that IRE1-dependent signaling is functionally important for airway inflammation [28,29,30], our research has focused on this UPR pathway. IRE1 exists in two isoforms in mammals, α and β, which are only 39% identical at the amino acid level [31]. IRE1α is expressed in all tissues, but IRE1β is only expressed in mucous cells of the respiratory and GI tracts [32]. We have shown that IRE1α activation is required for cytokine production during inflammation of CF human airway epithelia [28] and CF human alveolar macrophages [30]. In contrast, activation of IRE1β is required for airway epithelial mucin production, and this function is specific for IRE1β vs. IRE1α [32].

IRE1 is an ER stress sensor that contains lumenal, transmembrane and cytoplasmic regions [31]. The cytoplasmic portion of the protein contains both kinase and endoribonuclease (RNase) domains which give rise to IRE1′s dual enzymatic activities. Increased ER stress triggers oligorimerization of IRE1α’s inactive monomers, leading to dimerization and activation of its cytoplasmic kinase domain, resulting in a conformational change that activates its C-terminal RNase [33]. Active IRE1α RNase is responsible for sequence-specific cleavage of an intron from the mRNA for the X-box binding protein-1 (XBP-1) [34,35,36]. The cleaved XBP-1 is subsequently re-ligated by RNA 2′,3′-cyclic phosphate and 5′-OH ligase allowing the formation of a frame-shifted, spliced XBP-1 (XBP-1s) [37,38]. Importantly, XBP-1s translates into a transcription factor that up-regulates the lipid constituents and protein folding capacity of the ER, which are necessary for ER/Ca^2+^ store expansion in inflamed airway epithelia exhibiting a robust inflammatory response [27,29]. In fact, we have previously shown that activation of IRE1α-dependent XBP-1s mediates airway cytokine production/secretion in models relevant to CF [22,27,28,29,30]. A review of the molecular pathways involved in IRE1α/XBP-1s signaling relevant to CF airway inflammation can be found in [27].

Our previous findings led to the notion that IRE1α is a novel therapeutic target for CF airway inflammation. Therefore, in the present study we tested whether pharmacological inhibition of IRE1α decreases cytokine production in a pre-clinical inflammatory model for CF consisting of mucosal exposure of well-differentiated primary cultures of F508del homozygous human bronchial epithelia (HBE) to supernatant of mucopurulent material (SMM) harvested from excised human CF lungs [22,39]. In addition, we tested whether a triple combination of CFTR modulators currently used in the clinic affects SMM-induced IRE1α activation coupled with cytokine production in presence or absence of pharmacological inhibition of IRE1α.

## 2. Results

### 2.1. IRE1α mRNA and Protein Expression Levels Are Up-Regulated in CF Human Airway Epithelia

**Figure 1:**
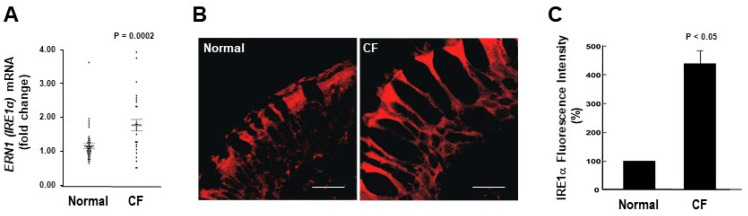
*ERN1*/*IRE1α* mRNA and protein expression is up-regulated in freshly isolated and native CF HBE. (**A**): Freshly isolated HBE from 41 normal and 28 CF lungs [mRNA fold change relative to TATA-box binding protein (TBP)]; dots correspond to individual lung samples. (**B**): Immunofluorescent stain of IRE1α expression in native HBE; representative images from 3 normal and 3 CF lungs; bars: 10 μm. (**C**): Compiled data from IRE1α expression in native normal and CF HBE (expressed as percentage of fluorescence intensity from normal native HBE). (**A**,**C**): Mean ± SEM. A and C: *p* values indicate the comparisons between CF vs. normal samples.

We first assessed the mRNA levels of *ERN1/IRE1α* in freshly isolated HBE from normal and inflamed CF airways. We found that *ERN1/IRE1α* mRNA expression is up-regulated in CF HBE (Figure 1A). We next evaluated by confocal microscopy whether the increased mRNA expression of *IRE1α* in freshly isolated CF HBE corresponded to an increased expression of IRE1α protein in native CF HBE. Figure 1B illustrates that IRE1α protein expression is up-regulated in native CF HBE as compared with normal HBE. Notably, in both normal and CF native HBE, the expression of IRE1α is polarized towards the apical domain of the epithelia (Figure 1B). Because IRE1α is an ER resident protein, and is a marker of this organelle, these findings are also in agreement with our previous observations that the ER network extends towards the apical domain of airway epithelia and is expanded in inflamed CF airways [26]. Quantification of the IRE1α immunostain confirmed the IRE1α up-regulation in CF vs. normal native HBE (Figure 1C).

### 2.2. The Up-Regulation of IRE1α and XBP-1s Found in Freshly Isolated CF HBE Is Reproduced in Primary Normal HBE Cultures Exposed to SMM

**Figure 2:**
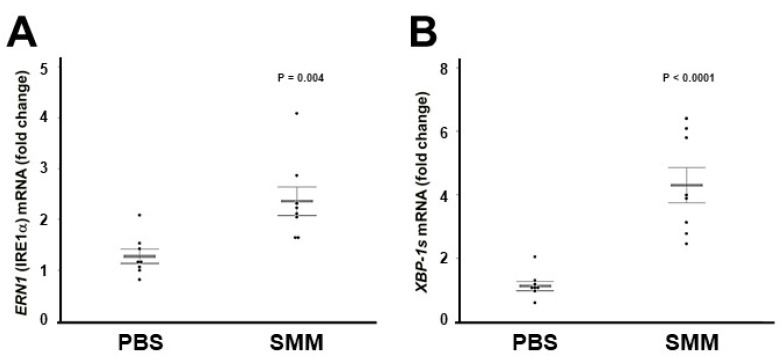
Mucosal exposure of normal HBE to SMM reproduces the up-regulation of *ERN1/IRE1α* and *XBP-1s* mRNA levels found in inflamed, freshly isolated or native CF HBE. *ERN1* (**A**) and *XBP-1s* (**B**) mRNA expression from PBS- or SMM-exposed cultures. Data are from 72 h mucosal HBE exposure to PBS or SMM, and relative to 18S mRNA. Data are shown as mean ± SEM. n = 8 normal lungs. *p* Values depict the comparisons between SMM- vs. PBS-exposed cultures.

We have reported that freshly isolated, inflamed CF HBE exhibit higher levels of XBP-1s [28]. These findings are associated with the up-regulation of IRE1α expression in freshly isolated or native CF HBE (Figure 1), and indicate that the activities of IRE1α kinase and RNase are increased in inflamed CF HBE. To address whether the increased levels of IRE1α and XBP-1s in CF HBE result from an acquired response to airway inflammation, we utilized a model developed in our laboratory consisting of exposure of normal HBE devoid of CFTR mutations to SMM from human CF airways [22,28,39,40]. SMM exposure promotes HBE inflammation and triggers IRE1α-dependent XBP-1s-induced ER expansion linked with increased cytokine production [28]. Well-differentiated primary cultures of normal HBE were exposed to mucosal PBS (control) or SMM, and the mRNA levels of *ERN1/IRE1α* and *XBP-1s* were evaluated after 72 h. SMM up-regulated *ERN1/IRE1α* mRNA expression (Figure 2A), and this effect was associated with increased *XBP-1s* mRNA levels (Figure 2B). These findings indicate that (a) exposure of normal HBE to SMM reproduces the up-regulation of IRE1α and XBP-1s found in inflamed, freshly isolated/native CF HBE (Figure 1; [28]) and (b) the increased expression of IRE1α and XBP-1s in CF HBE reflects an acquired response to the CF airway milieu.

### 2.3. The Small Molecule IRE1α Kinase and RNase Inhibitor KIRA6 (Kinase Inhibiting RNase Attenuating 6) Binds Directly to IRE1α Kinase

**Figure 3:**
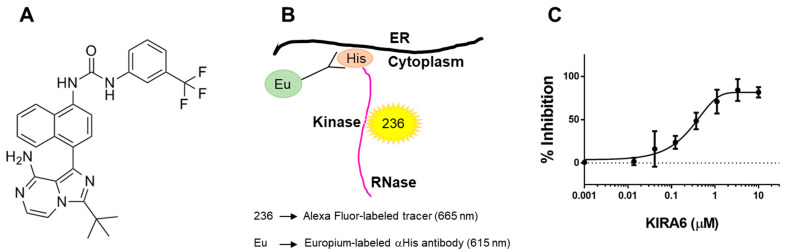
TR-FRET assay to evaluate the binding of KIRA6 to IRE1α kinase. (**A**): Structure of KIRA6 (CAS No: 1589527-65-0). (**B**): Schematic principle of the TR-FRET assay designed to evaluate KIRA6 binding to the kinase domain of full-length cytoplasmic IRE1α. (**C**): KIRA6 binds purified IRE1α kinase with an apparent *K*_d_ of 0.28 µM (GraphPad Prism). The FRET response signal (ratio 665nm/615nm) was normalized to 0% = vehicle and 100% = inhibition with 10 µM CTx-0294885 (positive control). Data represent mean ± SEM (n = 3 separate assays).

We evaluated the ability of the IRE1α kinase and RNase inhibitor KIRA6 [41] to inhibit IRE1α-dependent cytokine production. KIRA6 was selected because of its commercial availability and its type II kinase inhibitor profile. KIRA6 is a nanomolar ATP-competitive inhibitor that locks the IRE1α kinase in an inactive (DFG-out), monomeric state that results in an allosteric attenuation of the RNase activity [41]. Interestingly, opposing actions for type I versus type II kinase inhibitors on the IRE1α RNase have been previously described [42]. While type I inhibitors promote IRE1α oligomerization and RNase activation, type II inhibitors potentially block these actions [42]. Additionally, type II kinase inhibitors, such as KIRA6, tend to be more selective than type I inhibitors due to the DFG-out binding conformation allowing for more contacts with the catalytic site [43]. However, uncharacterized “off-target” activities cannot be excluded at higher concentrations. In fact, a report suggests that KIRA6 may also inhibit Kit kinase, but only at higher doses (*K*_d_ = 10.8 µM) [44]. The structural formula of KIRA6 is depicted in Figure 3A [45]. To confirm that KIRA6 binds directly to the kinase domain of full-length cytoplasmic IRE1α, we utilized a TR-FRET assay that measures the displacement of a fluorescent ligand, 236, from the isolated, His-tagged IRE1α kinase bound to a Europium-labeled anti-His antibody (ThermoFisher; Figure 3B). When 236 binds the ATP binding site of the IRE1α kinase, there is a high degree of FRET from the Europium to the Alexa Fluor™, resulting in a high ratio of Alex Fluor to Europium signal. By design, displacement of 236 binding by an IRE1α kinase inhibitor causes the FRET signal to diminish. KIRA6 dose-dependently displaced the binding of 236 from the IRE1α kinase in our TR-FRET assay, with an apparent *K*_d_ of 0.28 µM (Figure 3C).

### 2.4. Proof-of-Concept That Pharmacological Inhibition of IRE1α Kinase and RNase Blocks XBP-1s Expression Coupled to Cytokine Production in CF HBE

**Figure 4:**
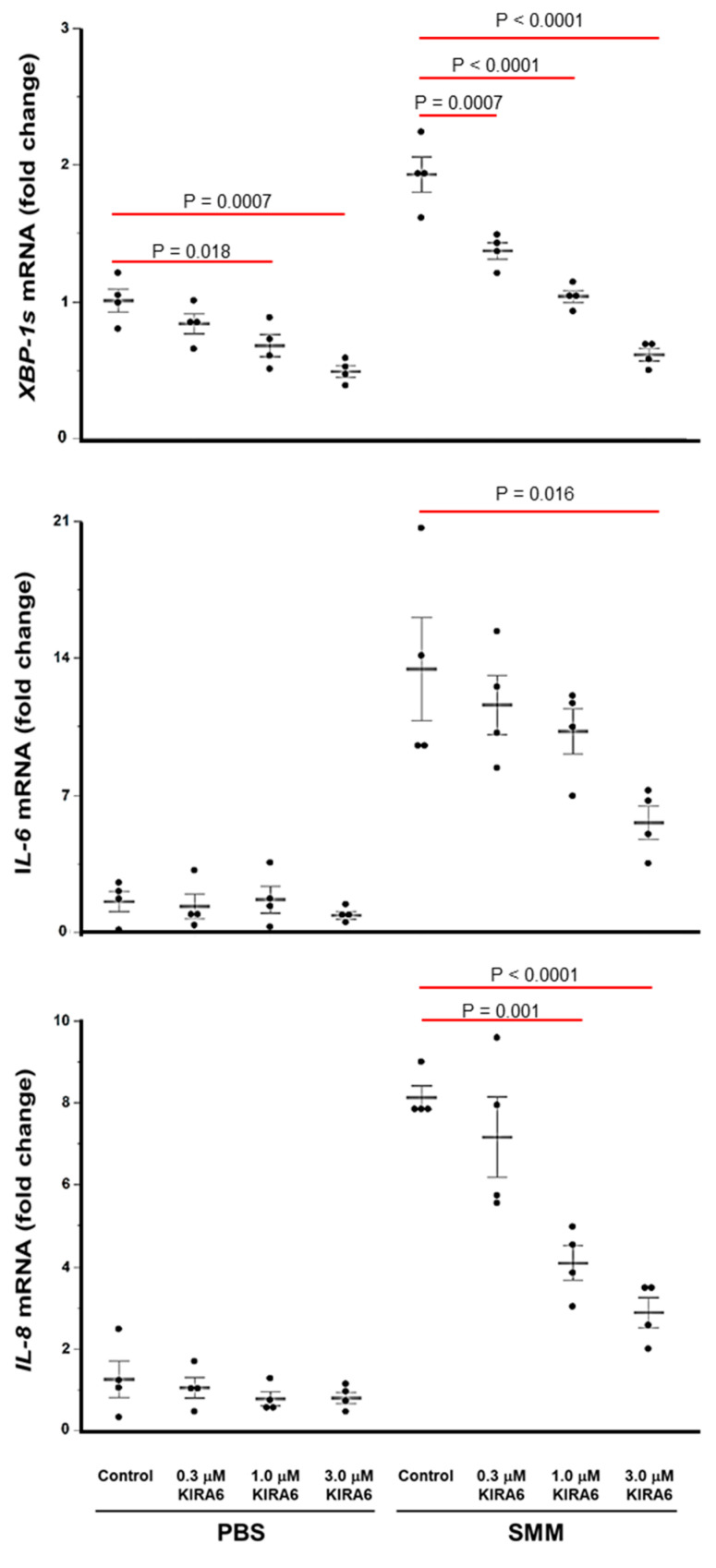
Inhibition of IRE1α kinase and RNase with KIRA6 blunts, in a dose-dependent manner, SMM-up-regulated *XBP-1s*, *IL-6* and *IL-8* mRNA levels in primary cultures of F508del HBE. Data are expressed as fold change over 18S, and shown as mean ± SEM. n = 4 CF lungs. *p* Values depict statistical differences between specific groups.

Since binding of KIRA6 to the IRE1α kinase should result in attenuation of the IRE1α RNase-dependent generation of XBP-1s [41,42], we conducted studies in primary F508del homozygous HBE cultures mucosally exposed to PBS or SMM, in presence or absence of various doses of KIRA6, to evaluate whether KIRA6 can attenuate IRE1α-driven cytokine production. We first evaluated the mRNA levels of *XBP-1s*, interleukin *(IL)-6* and *IL-8* after 24 h with the various treatments. In agreement with previous studies [22,27,28,29,30], SMM up-regulated *XBP-1s* mRNA expression, and this response was associated with SMM-induced up-regulation of the mRNA levels of the cytokines *IL-6* and *IL-8* (Figure 4). However, SMM-increased *XBP-1s*, *IL-6* and *IL-8* mRNA levels were blunted, in a dose-dependent manner, by KIRA6 (Figure 4). In PBS-exposed CF HBE, KIRA6 dampened the *XBP-1s* mRNA levels, but it did not promote inhibition of *IL-6* or *IL-8* mRNA expression (Figure 4).

**Figure 5:**
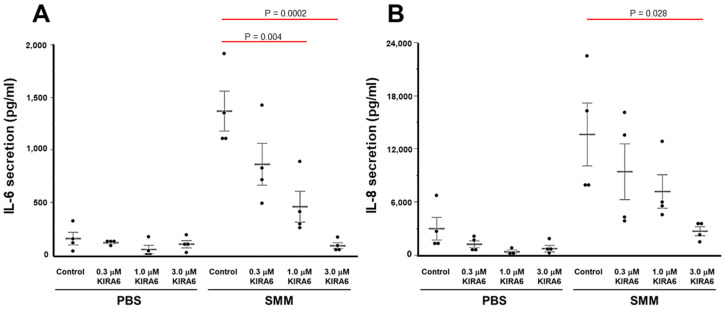
KIRA6 blunts, in a dose-dependent manner, SMM-increased IL-6 and IL-8 secretion in primary cultures of F508del HBE. IL-6 (**A**) and IL-8 (**B**) secretion is expressed as pg cytokine secretion/mL of culture media, and shown as mean ± SEM. n = 4 CF lungs. *p* Values depict statistical differences between specific groups.

We next evaluated whether the inhibitory effect of KIRA6 on SMM-up-regulated IL-6 and IL-8 mRNA corresponded to inhibition of SMM-increased IL-6 and IL-8 secretion. Primary F508del homozygous HBE cultures were mucosally exposed to PBS or SMM for 72 h, in presence or absence of various doses of KIRA6. SMM increased the secretion of IL-6 and IL-8 (Figure 5A,B), in agreement with our previous findings [22,27]. KIRA6 dose-dependently decreased these responses (Figure 5A,B), while in PBS-exposed CF cultures, KIRA6 did not significantly inhibit IL-6 or IL-8 secretion (Figure 5A,B).

### 2.5. A Triple Combination of CFTR Modulators Does Not Blunt SMM-Increased XBP-1s and Cytokine Production in CF HBE

**Figure 6:**
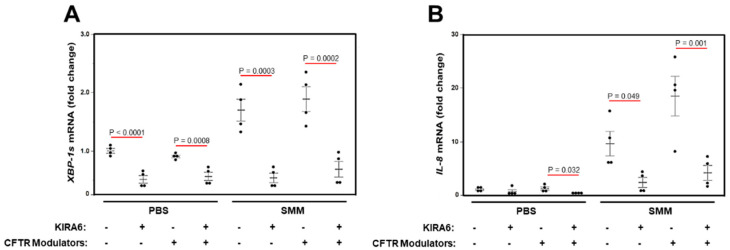
A triple combination of CFTR modulators does not decrease SMM-promoted generation of XBP-1s coupled to IL-8 production in F508del/F508del HBE. (**A**,**B**): Cultures were treated for 48 h with mucosal PBS or SMM, and serosal vehicle (DMSO) or 3 µM VX-661 + 2 µM VX-445 + 1 µM VX-770, in the absence or presence of 3 µM KIRA6. Data are expressed as fold change of *XBP-1s* (**A**) or *IL-8* (**B**) mRNA levels over 18S, and shown as mean ± SEM. n = 4 cultures from 2 CF lungs. *p* Values depict statistical differences between specific groups.

Trikafta^®^ [triple CFTR modulator therapy with elexacaftor (VX-445), tezacaftor (VX-661), and ivacaftor (VX-770)] has been approved by the FDA for the treatment of CF patients aged 12 years and older who have at least one copy of the F508del mutation. We tested the effect of this triple therapy on SMM-up-regulated *XBP-1s* and *IL-8* mRNA levels in primary cultures of homozygous F508del HBE. While the triple therapy effectively rescued F508del CFTR (data not shown), it did not decrease basal (PBS-treated) or SMM-up-regulated *XBP-1s* and *IL-8* mRNA levels (Figure 6A,B). Moreover, while KIRA6 inhibited SMM-increased *XBP-1s* and *IL-8* mRNA levels, the triple CFTR modulator therapy had no effect on the inhibitory action of KIRA6 (Figure 6A,B). These findings indicate that this triple combination of CFTR modulators currently used in the clinic does not blunt SMM-promoted generation of XBP-1s coupled to IL-8 production, whereas inhibition of IRE1α with KIRA6 does. In addition, our data further suggest that airway inflammation, but not CFTR dysfunction, is responsible for increased CF airway epithelial cytokine production coupled to activation of IRE1α/XBP-1s signaling.

## 3. Discussion

Although the chronic inflammatory status of CF airways is associated with increased morbidity and mortality [46], conventional anti-inflammatory therapies have not proven ideal and possess adverse effects. For instance, the use of oral corticosteroids may lead to glucose intolerance, growth retardation and cataracts. Therefore, the CF Foundation recommends against the chronic use of oral corticosteroids to improve lung function in CF children [47,48]. Moreover, oral corticosteroids do not improve lung function in adults with CF, and there is concern that their chronic use may lead to steroid-related diabetes mellitus and osteoporosis. Similarly, because inhaled corticosteroids have not proven beneficial towards improving lung function or reducing hospitalizations, the CF Foundation does not recommend their use in CF patients who do not suffer from asthma or allergic bronchopulmonary aspergillosis [47,48]. Nonsteroidal anti-inflammatory drugs, e.g., ibuprofen, are better tolerated. However, although oral ibuprofen therapy has been associated with a slower decline in FEV_1_, particularly in younger CF patients [49], and chronic ibuprofen use has been linked to improved survival [50], this therapy can lead to adverse effects such as gastrointestinal bleeding and ulcers and kidney injury. The macrolide antibiotic azithromycin has anti-inflammatory properties [51], can improve lung function [52] and is recommended for CF patients of six years and older chronically infected with *P. aeruginosa* [53]. However, its long-term treatment may result in macrolide-resistant infection. The recently approved CFTR modulators are showing clinical benefits. However, solid data on their possible anti-inflammatory effect are not available yet. Based on these considerations, there is a clear unmet medical need for new therapies for CF airways inflammation.

Previous studies indicated that CF airway inflammatory responses are linked with activation of the IRE1α pathway. Specifically, the levels of XBP-1s are increased in freshly isolated CF vs. normal airway epithelia [28]. This alteration can be reproduced in translational models relevant to CF airways disease, further linking activation of IRE1α to CF airway inflammatory responses [27,28]. For instance, we have shown that exposure of airway epithelia to SMM up-regulates the levels of XBP-1s and this response is linked with an increased secretion of cytokines [22,27]. Furthermore, while XBP-1s levels are increased in inflamed CF HBE, they revert to normal levels in primary CF HBE cultures that have lost their excessive inflammatory response [54]. Notably, these studies also indicated that the increased levels of XBP-1s found in inflamed CF airway epithelia are not linked to CFTR mutations [22,27,54]. In addition, the functional role of IRE1α/XBP-1s signaling has been subsequently extended to primary cultures of CF alveolar macrophages by showing that they exhibit an exaggerated production of cytokines, which is mediated by IRE1α-dependent XBP-1s [30]. A previous study from our group evaluated the mRNA levels of *IRE1α (ERN1)* and *IRE1β (ERN2)* by RNAscope in proximal to terminal airway epithelia from normal vs. CF subjects [55]. Our findings indicated that while the expression of IRE1α and IRE1β is increased in CF, the tissue expression of IRE1α is more generalized, in contrast to the restricted expression of IRE1β to the epithelial layer. Together, these previous findings led to the notion that inhibition of IRE1α might provide a new therapeutic strategy for controlling the excessive inflammation of CF airways by targeting the activity of this ER stress protein in multiple cell types.

The current paradigm is that the IRE1α kinase is an allosteric modulator of the IRE1α RNase activity and, thus, regulates the levels of XBP-1 mRNA splicing [33]. When ER stress occurs resulting from, e.g., increased ER levels of newly synthesized unfolded cytokine proteins, it causes IRE1α dimerization and trans-autophosphorylation of the cytoplasmic kinase domain, leading to a conformational change that activates the RNase and allows each RNase dimer to dock a XBP-1 mRNA hairpin loop, promoting the formation of the transcription factor XBP-1s [33,56]. While RNase inhibitors have been described, such as the aldehyde-containing 4-methyl umbelliferone 8-carbaldehyde (4µ8C) and STF-083010, that can prevent this docking, thereby reducing XBP-1 splicing and downstream XBP-1s-dependent signaling [36,57,58], micromolar potencies and ‘druggability’ issues limit the clinical utility of these covalent modifiers.

The majority of IRE1α kinase inhibitor ligands that have been reported, such as the type I ATP competitive kinase inhibitors CTx-0294885 [59] and Sunitinib, promote IRE1α RNase dimerization, leading to increased RNase-directed mRNA splicing of XBP-1 [33]. Feldman et al. have identified 15 of these IRE1α RNase activating, kinase inhibiting ligands from a Selleckchem kinase inhibitor library [60]. However, like CTx-0294885, these tend to be non-selective for other kinases and are not suitable for comparison experiments pairing dual kinase/RNase inhibitors with kinase inhibitors that activate the RNase. Far less common are the IRE1α kinase inhibitors termed KIRAs [42,60]. These inhibitors block both the kinase and RNase activities of IRE1α, likely by preventing dimerization, and have more favorable selectivity profiles [42,60].

The present study tested the premise that pharmacological inhibition of the IRE1α kinase and RNase with KIRA6 could be used to ameliorate the robust inflammatory response characteristic of CF airways. Our findings can be summarized as follows. (1) IRE1α expression is up-regulated in freshly isolated and native CF bronchial epithelia (Figure 1). (2) The increased IRE1α levels found in CF airway epithelia can be reproduced in vitro in normal HBE exposed to SMM (Figure 2), indicating that this is an acquired response induced by the inflammatory CF airway milieu. Further, (3) inhibition of IRE1α kinase and RNase activities with KIRA6 blunts SMM-increased cytokine production in CF HBE (Figure 4 and Figure 5). The pre-clinical model consisting of primary cultures of homozygous F508del CF HBE grown at air-liquid interface [22] and exposed to SMM harvested from the airways of excised human CF lungs [22,28,39,40] was utilized because it recapitulates several aspects of CF airway epithelial inflammation [22,27,28,39,40]. SMM provides an “all-inclusive” stimulus representative of the CF airway milieu because it contains (1) products from bacteria, factors from neutrophils, including neutrophil elastase, lysozyme, cathepsin G, and MMP9 [39]; (2) secretory products from airway epithelia and macrophages, including cytokines [39]; and (3) hundreds of peptides, mucins and purines [61]. The inflammatory composition of SMM is reproducible from patient to patient [39]. Because the airway epithelia of CF patients are exposed to the combination of all these inflammatory factors, the use of SMM is a superior approach vs. the use of single inflammatory factors for testing the efficacy of small molecule inhibitors of IRE1α as anti-inflammatory therapeutics.

While novel anti-inflammatory therapies for CF lung disease devoid of adverse effects are necessary, care needs to be exercised to avoid decreasing the inflammatory response beyond a minimal level required for controlling bacterial infections [62]. An important aspect of the present study is that KIRA6 did not decrease the levels of cytokine production/secretion beyond the basal levels measured in the absence of SMM (Figure 4 and Figure 5). Instead, our findings indicated that KIRA6 blunted only the excessive inflammatory component resulting from SMM exposure, which under chronic, persistent conditions, is expected to promote damage of CF airways.

An important aspect of our study is the finding that the triple CFTR modulator therapy consisting of VX-445, VX-661, and VX-770 did not blunt basal or SMM-up-regulated XBP-1s coupled with increased IL-8 production, whereas KIRA6 did (Figure 6). In agreement with these observations, recent studies from our group demonstrated that different combinations of CFTR modulators (e.g., VX-661, VX-661 + VX-770, VX-809, VX-809 + VX-770, VX-659, VX-659 + VX-770, VX-661 + VX-659, and VX-661 + VX-659 + VX-770) were devoid of an anti-inflammatory effect on SMM-exposed F508del HBE cultures [63]. Considering the contribution of CF airway epithelia to the robust cytokine levels of CF airways [27], our data indicate that a high CF airway inflammatory status may remain problematic in presence of currently available CFTR modulators. Consistent with this view, airway inflammation in CF patients with the G551D mutation was not reduced by the CFTR potentiator VX-770 in spite of improvements in other clinical parameters [64,65], although decreases in airway inflammation were reported in another study [66]. Our findings lead to the notion that CFTR modulators may be used in combination with IRE1α inhibitors to simultaneously achieve airway hydration and decreased airway inflammation.

It should be noted that IRE1α can also act in a XBP-1s-independent manner. For instance, ER stress-induced IRE1α activation can recruit tumor necrosis factor receptor associated factor 2 (TRAF2) to its cytoplasmic kinase domain [67]. TRAF2 activates protein kinases involved in immunity and inflammation, including apoptosis signal-regulating kinase-1 (ASK1), which in turn activates cJUN NH_2_-terminal kinase (JNK) and p38 mitogen-activated protein kinase (MAPK) [68]. IRE1α kinase can also activate NF-κB via TRAF2 [69,70]. Because activation of these pathways can mediate inflammatory responses [27], we speculate that activation of the IRE1α kinase may also lead to cytokine production in CF airways independent of IRE1α RNase activation. Future studies will be necessary to tease out the relative contributions of the IRE1α kinase via XBP-1s-dependent vs independent regulation of inflammatory responses in CF airways. Further, the selectivity of KIRA6 may need further refinement, as a new report found KIRA6 can induce a marker of autophagy in Neuro2a cells independent of IRE1α [71]. Nevertheless, our present study suggests that IRE1α plays a significant role in the excessive cytokine production of CF airways, and that small molecule inhibition of IRE1α kinase with a compound that allosterically also blunts RNase activity may have clinical utility.

**Figure 7:**
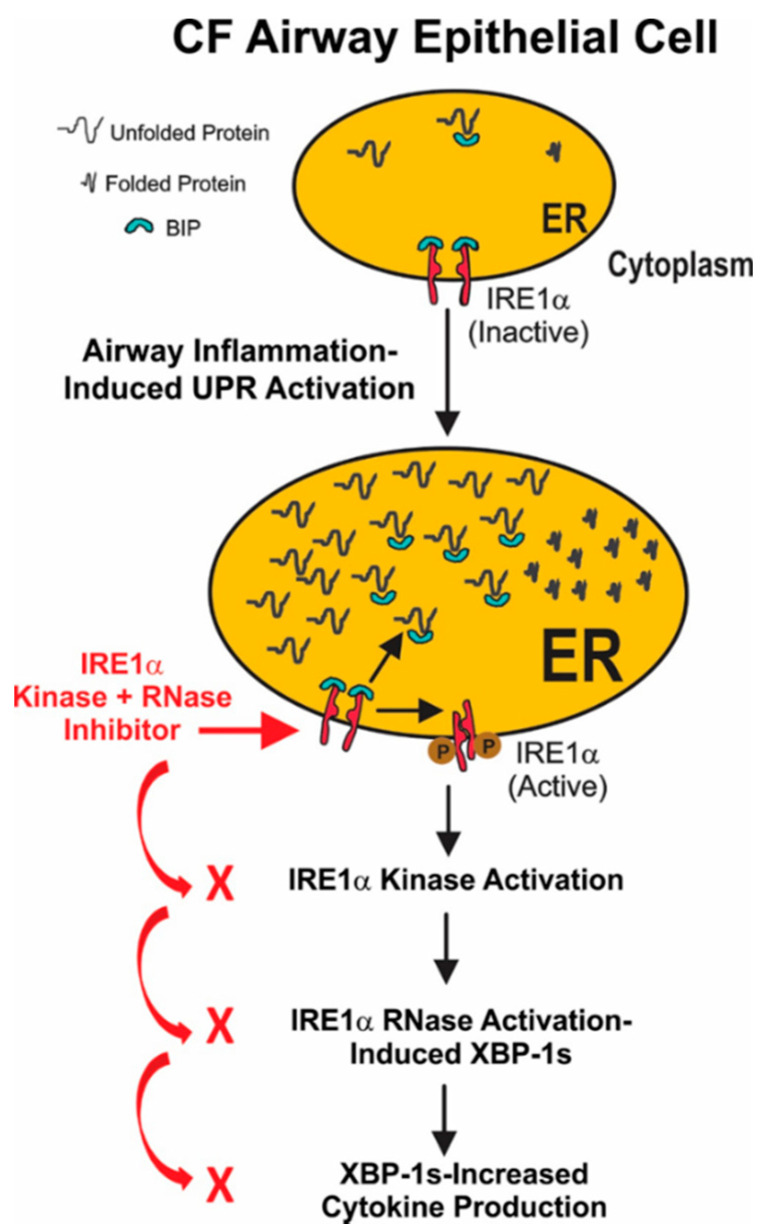
Model for IRE1α activation-dependent cytokine production in inflamed CF airway epithelia. Airway inflammation induces ER stress and triggers the unfolded protein response in CF airway epithelia. The current notion is that under normal conditions the immunoglobulin binding protein (BIP; also known as glucose-regulated protein 78, GRP-78) binds to the ER lumenal domain of IRE1α, repressing its activation. However, following ER stress, BIP dissociation from IRE1α results in activation of the IRE1α kinase and RNase, leading to the mRNA splicing if XBP-1 (XBP-1s). The resulting XBP-1s is a transcription factor that up-regulates cytokine production in inflamed CF airway epithelia. Inhibition of IRE1α kinase and RNase activities with a small molecule like KIRA6 decreases XBP-1s levels, blunting XBP-1s-mediated cytokine production, thereby ameliorating CF airway inflammation.

In conclusion, our findings lead to the model depicted in Figure 7. Airway inflammation induces ER stress and triggers the unfolded protein response in CF airway epithelia. This results in activation of the IRE1α kinase and RNase activities, leading to the mRNA splicing of XBP-1. The resulting XBP-1s is a transcription factor that up-regulates cytokine production in inflamed CF airway epithelia. Our data provide the proof-of-concept that small molecule IRE1α kinase inhibitors like KIRA6 that allosterically reduce RNase-dependent XBP-1 mRNA splicing may lead to a novel approach for treating the excessive inflammation characteristic of CF airways.

## 4. Materials and Methods

### 4.1. Tissue Harvesting

Human bronchial epithelial (HBE) cells were obtained from normal and CF bronchi according to protocols approved by the University of North Carolina Biomedical Institutional Review Board (study #18-1031, approved on 4/20/2018). Both cell types were isolated from excised lungs (e.g., for normal cells, lungs rejected for transplant by local and distant organ procurement agencies; for CF cells, lungs excised at the time of a transplant). Normal lungs originated from subjects with no history of inflammatory or infectious disease known to affect the respiratory airways. For some studies, sections from paraffin-embedded native normal and CF bronchial tissues were used. Cells and native tissues were provided by the Tissue Procurement and Cell Culture Core from the Cystic Fibrosis Research Center at the University of North Carolina at Chapel Hill as previously described [22,26,39].

### 4.2. Supernatant of Mucopurulent Material (SMM) from CF Airways

Mucopurulent material was harvested from the airway lumens of excised human CF lungs as previously reported [22,26,39], and was provided by the UNC CF Center Tissue Procurement and Cell Culture Core. Briefly, the mucopurulent material was centrifuged at 100,000 rpm for 60 min at 4 °C and the resulting supernatant (SMM) was filtered with a 0.2 µm filter. SMM was pooled from several CF lungs, aliquoted into single use tubes and stored at −80 °C.

### 4.3. Cell Culture and Treatments

Primary normal HBE (for studies mimicking the CF phenotype, as described in Figure 2) or F508del homozygous CF HBE were grown for 28 days and studied as polarized, well-differentiated cultures under airway liquid interface conditions, as previously described [22,26,39]. Cultures were exposed to 30 μL mucosal PBS or SMM for various amounts of time in presence or absence of the IRE1α kinase and RNase inhibitor, Kinase Inactivating RNase Attenuating 6 (KIRA6, CAS No.: 1589527-65-0 [41]; EMD Millipore, Burlington, MA, USA), which was added to the serosal compartment, as detailed in the figure legends.

To evaluate the effect of a triple combination of CFTR modulators on SMM-up-regulated XBP-1s coupled to IL-8 production, primary cultures of F508del/F508del HBE were treated for 48 h with mucosal PBS or SMM, and serosal vehicle (DMSO) or 3 µM tezacaftor (VX-661, Selleck Chemicals) + 2 µM elexacaftor (VX-445, MedChemExpress) + 1 µM ivacaftor (VX-770, Selleck Chemicals, Houston, TX, USA), in the absence or serosal presence of 3 µM KIRA6.

### 4.4. Immunofluorescence and Quantification of IRE1α Expression

The immunostaining of IRE1α in 4% paraformaldehyde-fixed and deparaffinized native bronchial epithelial sections from normal and CF lungs was performed according to a modification of our previous method [26]. All incubations and washes between steps were performed with PBS. Sections were permeabilized with 1% Triton X-100 for 10 min at room temperature, rinsed three times, blocked with 3% bovine serum albumin for 30 min at 37 °C, and washed three times. Sections were subsequently incubated overnight at 4 °C with a goat polyclonal IRE1α primary antibody (1:100 dilution; Santa Cruz Biotechnology, Dallas, TX, USA), followed by three washes and incubation with a Texas Red-labeled donkey anti-goat antibody (1:200 dilution; Jackson Immunoresearch Laboratories, West Grove, PA, USA) for 1 h at room temperature. Sections were subsequently washed three times and mounted with a coverslip. As a control, the primary antibody was omitted. The fluorescent signals were studied by laser confocal microscopy (Leica, model TCS 4D; PL APO 63×/1.20 mm water lens) in the XY scanning mode. The quantification of the fluorescence intensity of labeled IRE1α was performed according to a previous method [26] utilizing the MetaMorph^®^ Microscopy Automation and Image Analysis software. The same acquisition parameters (e.g., laser power, contrast, brightness and pinhole value) were employed to acquire the images from native normal and CF bronchial epithelia in experiments performed on the same day.

### 4.5. Real Time Polymerase Chain Reaction (RT-PCR)

RT-PCR was used to evaluate *IRE1α* (*ERN1*), *XBP-1s*, *IL-6* and *IL-8* mRNA levels, as previously described [30]. Briefly, RNA was recovered from cultures lysed in TRI reagent using the Zymo Direct-zol mini kit (R2052, Zymo Research, Irvine, CA, USA), and cDNA was made from 500 ng of RNA using the iScript cDNA synthesis kit (1708841, Bio-Rad, Hercules, CA, USA). cDNA was then diluted 1:20 and 4 μL were used with 5 μL SsoAdvanced Universal Probes Supermix (1725282, Biorad, Hercules, CA, USA), 0.5 μL of one Taqman Probeset (ThermoFisher Scientific, Waltham, MA, USA; IL6: Hs00174131_m1, IL8: Hs00174103_m1, XBP1s: Hs03929085_g1, TBP: Hs00427620_m1, 18s: Hs99999901_s1), and DNase-free water to 10 μL total volume. The samples were run on a Quant-6 RT-PCR machine with the following protocol: 95 °C for 20 s, followed by 40 cycles of 95 °C for 20 s and 60 °C for 20 s. For quantification of mRNA levels, the ΔC_T_ value was produced by subtracting the C_T_ value of the housekeeping gene (18 s or TBP) from the gene of interest, as reported [30].

### 4.6. Expression of the Cytoplasmic Domain of IRE1α

The soluble cytoplasmic portion of human IRE1α containing both the serine/threonine kinase and RNase domains (residues 547-977 of accession number NP_001424) was cloned and expressed. Briefly, IRE1α was expressed as N-terminal His-tagged protein in Sf9 cells using baculovirus produced from pFastBacHT (Life Technologies, Carlsbad, CA, USA). Cells were harvested after 72 h and cell pellets were stored at −80 °C until purification.

### 4.7. Purification of Cytoplasmic IRE1α for Kinase Assays

Cell pellets were thawed, resuspended, and lysed in ‘cytobuster’ (EMD Millipore) containing protease (EDTA-free) and phosphatase inhibitor cocktails (Pierce) and 30 mM imidazole, pH 8.0. The resulting cell lysates were clarified and affinity purified on HisTrapFF nickel columns (GE Healthcare) using an AKTA FPLC (GE Healthcare, Marlborough, MA, USA). Bound protein was eluted in a linear gradient of elution buffer (50 mM sodium phosphate buffer, pH 7.2, 500 mM NaCl, 500 mM imidazole). Peak fractions were run on SDS-PAGE gels and analyzed by Coomassie staining. IRE1α containing fractions were pooled and concentrated in Amicon Ultra 30K cut off concentrators (EMD Millipore) and buffer was exchanged into 25 mM HEPES, pH 7.5, 150 mM NaCl, 2 mM DTT, 5% glycerol. Protein concentration was determined by Bradford assay. Protein was aliquoted and stored at −80 °C, with additional glycerol (25% final) added for longer term storage. This expression and purification approach has been successfully used to study IRE1α kinase activity in vitro [58,60,72].

### 4.8. Time Resolved—Fluorescence Resonance Energy Transfer (TR-FRET) IRE1α Kinase Assay

We developed a 384 well formatted TR-FRET assay using LanthaScreen reagents (ThermoFisher) to evaluate the binding of the IRE1α inhibitor KIRA6 (EMD Millipore or MedChemExpress, Monmouth Junction, NJ, USA) to the purified full-length cytoplasmic domain of IRE1α. We utilized a displacement assay format with a Europium-labeled antibody that recognizes the N-terminal His tag on the IRE1α cytoplasmic protein as an energy donor, and an Alexa Fluor™-labelled kinase ligand, 236, as the energy acceptor (ThermoFisher). CTx-0294885, a non-selective, broad spectrum kinase inhibitor, was used as a positive control for the assay (MedChemExpress). After incubating the assay mix consisting of 20 nM his-tagged IRE1α, 2 nM Europium anti-his antibody, and 40 nM 236 tracer, the TR-FRET signal ratio (665 nm/615 nm) was collected using a Perkin Elmer EnVision^®^ plate reader. Eight point compound dose curves diluted in half logs starting at 10 µM were utilized to generate the data.

### 4.9. Cytokine Secretion

Quantification of IL-6 and IL-8 secretion into cultured media was performed by standard ELISA measurements in duplicate samples, and utilized reagents from R&D Systems as we previously published [22]. These cytokines were chosen because they are predominant cytokines present in CF airways [39]. Secretion of IL-6 and IL-8 resulting from the different treatments was evaluated after 72 h.

### 4.10. Statistical Analyses

For the native tissue and HBE studies, we performed either unpaired Student’s T-test or one-way ANOVA, including standard (parametric) methods and the Tukey–Kramer multiple comparisons test [32] or the Dunnett test, utilizing the JMP Software from SAS.

Data from bar graphs or dot plots represent the mean ± SEM and statistical significance was defined with *p* < 0.05. For the TR-FRET kinase binding assays, compounds were tested in 8-point dose curves with half-log dilutions from a top concentration of 10 µM. GraphPad Prism6 was used to calculate 4-parameter fits of response data normalized to 100% inhibition equal to the amount of FRET signal displaced by 10 µM CTx-0294885 (positive control for maximal IRE1α kinase binding), and 0% equal to the vehicle control (1% DMSO).

## Data Availability

Not applicable.
